# Guidelines for cadaver dissection in education and research of clinical medicine (The Japan Surgical Society and The Japanese Association of Anatomists)

**DOI:** 10.1007/s12565-022-00665-8

**Published:** 2022-05-24

**Authors:** Toshiaki Shichinohe, Takashi Kondo, Hiroshi Date, Masako Hiramatsu, Satoshi Hirano, Chizuka Ide, Toshihiko Iwanaga, Yoshimitsu Izawa, Akio Kikuta, Eiji Kobayashi, Yoshiro Matsui, Yutaka Nohara, Takanori Shibata, Yasuhiro Shirakawa, Takane Suzuki, Haruo Takahashi, Hiroshi Taneichi, Toshiyuki Tsurumoto, Yasuo Uchiyama, Masahiko Watanabe, Hiroyuki Yaginuma, Kumiko Yamaguchi, Kazunari Yoshida

**Affiliations:** 1 The Japan Surgical Society, Guidelines Review Committee and CST Promotion Committee, World Trade Center Building South Tower 11F, 2-4-1, Hamamatsu-cho, Minato-ku, Tokyo, 105-5111 Japan; 2 The Japanese Association of Anatomists, c/o Oral Health Association, Komagome TS Bldg., 1-43-9, Komagome, Toshima-ku, Tokyo, 170-0003 Japan

**Keywords:** Guideline, Ethical code, Human body donation, Cadaver surgical training, Postgraduate education

## Abstract

This article translates the guidelines for cadaver surgical training (CST) published in 2012 by Japan Surgical Society (JSS) and Japanese Association of Anatomists from Japanese to English. These guidelines are based on Japanese laws and enable the usage of donated cadavers for CST and clinical research. The following are the conditions to implement the activities outlined in the guidelines. The aim is to improve medicine and to contribute to social welfare. Activities should only be carried out at medical or dental universities under the centralized control by the department of anatomy under the regulation of Japanese law. Upon the usage of cadavers, registered donors must provide a written informed-consent for their body to be used for CST and other activities of clinical medicine. Commercial use of cadavers and profit-based CST is strongly prohibited. Moreover, all the cadaver-related activities except for the commercial-based ones require the approval of the University's Institutional Review Board (IRB) before implementation. The expert committee organized at each university for the implementation of CST should summarize the implementation of the program and report the details of the training program, operating costs, and conflicts of interest to the CST Promotion Committee of JSS.

## Introduction

Cadaver surgical training (CST) is popular overseas as a means to secure medical safety by training surgeons to be more skillful. In Japan, the Postmortem Examination and Corpse Preservation Act (PECP) permits us to dissect human cadavers for medical or dental education and research under specified conditions. However, due to the PECP’s lack of criteria on the use of cadavers for surgical training and research, CST has not spread across Japan. Such ambiguity under the law prevents us from improving our surgical skills. To address this issue, we formulated the aim of CST guidelines to set out rules and concepts to secure social legitimacy in implementing CST. This aligns with PECP and thus permits us to conduct CST in accordance with the administrative procedures and rules. This legitimates the usage of cadaver as “socially acceptable” so as not to be penalized for “destruction of corpses” as stated under Article 190 of the Penal Code.

We initiated a research project entitled “The Study of Future Training Systems of Surgical Skills and Procedures” in fiscal year (FY) 2008, with financial support from the Ministry of Health, Labour and Welfare (MHLW). The project involved an in-depth survey among 24 medical associations related to surgery with the aim of looking into their surgical skill training perspectives. Their unanimous answers informed us that the implementation of CST is needed as it is a valuable program in training surgeons to develop more meticulous skills. Particularly, CST is indispensable for the regions in situ where such exercises are useful due to their complicated structures, and for those showing a great range of differences from the animal models (Study of Future Training Systems of Surgical Skills and Procedures 2009).

Subsequently, another program called “The Study of Effective Surgical Training Systems” was conducted in FY 2009 as an MHLW research program. In this program, we conducted a survey among the members of the department of surgery and its related divisions (including oral surgery) in the university hospitals, and the department of anatomy in Japanese medical and dental schools (Study of Effective Surgical Training Systems 2010). Of the respondents, 87% of the surgeons reconfirmed the earlier result, stating that CST was useful for understanding complicated three-dimensional (3D) structures. Therefore, it is indispensable for technical training. Similarly, almost 94% of anatomists recognized the importance of current trends in conducting CST. In addition, 42 out of the 99 departments of anatomy reported that they had already used cadavers for purposes other than undergraduate anatomical training. They reported in detail the actual contents and background of the program that led them to use cadavers for clinical training and research. They further emphasized that CST was conducted de facto as part of undergraduate education and research on gross anatomy, while being compliant with the PECP. For example, they took special precautions such as notifying the donor of what CST is and obtaining their informed consent (IC). Based on the above-mentioned background, we affirm the value of CST in achieving higher surgical skills, assuring medical safety, and contributing greatly to the welfare of Japanese society. However, as its implementation entails legal and ethical challenges that must be addressed, an MHLW research project in FY 2010 entitled “The Study of Effective Surgical Training Systems” led to the drafting of a guideline as an official research report (Study of Effective Surgical Training Systems 2011; Comments of the Association of Anatomists on the Guidelines for Human Cadaver Dissection in Clinical Medical Education and Research 2012). Taking these into consideration, we, the members of the Japan Surgical Society (JSS) and the Japanese Association of Anatomists (JAA), on April 20, compiled and publicly issued the “Guidelines for Cadaver Dissection in Education and Research of Clinical Medicine (Guideline 2012),” after reaching a consensus through several rounds of discussions with authorities, associations, and organizations. The 2012 Guidelines uphold Article 190 of the Penal Code and PECP.

The aim of the guidelines is to define the terms and conditions for medical doctors and dentists to engage in CST under the current Japanese law. This further enables CST to become a part of authentic undergraduate education and research programs without provoking arguments that could become an obstacle to its realization. After the implementation of the guidelines, each university is required to launch a specialist committee upon obtaining consent from the relevant institutional research board (IRB), and organize a core team to implement CST.

Moreover, the JSS CST Promotion Committee (JSS CST-PC), an official committee organized by the JSS, JAA, and other related societies, established after the guidelines were published, will be proactive in dealing with issues related to its implementation, such as potential changes in social recognition and atmosphere, medical circumstances, and amendments of related legal regulations.

## Cadaver surgical training: aims and demands for expertise

Rising public interest in medical safety in recent years has required doctors to have sufficient clinical on-the-job training (OJT) experience and intensive practice with educational devices and model animals before moving on to real patients. However, there are limited opportunities to go through the state-of-the-art surgical operations with high-grade difficulty. Moreover, medical educational devices and model animals cannot provide us with real images when dealing with special cases, especially for structures with highly complex 3D relationships or lacking similar anatomical framework in model animals. In other countries, to overcome these disadvantages, cadavers are used for training and improving surgical skills. In Japan, we made the guidelines public in 2012, followed by the WHLW grant program entitled, “Practical Training Project for Improving Surgical Procedures” (PTPSP) in the same year. According to JSS CST-PC, 16 institutions launched CST as of 2017.

Cadavers that are used in clinical education and research have various purposes, such as postgraduate and lifelong education, basic learning to mastering high-level surgical skills, and research and development of innovative surgical maneuvers and medical devices (as shown in Table 1). CST is a superb methodology that enables us to observe or gain detailed knowledge on the structures that cannot be seen in depth during routine surgery because of the risk of injury or death.

**Table 1:** Examples of cadaver use in medical education and research

1. Acquisition of basic medical skills
Use of cadavers by doctors-in-training to acquire the anatomical knowledge indispensable for safely performing the medical techniques
2. Acquisition of basic surgical and interventional skills
Use of cadavers for learning essential surgical and interventional techniques in lieu of on-the-job training (OJT) or training on animal subjects
3. Acquisition of surgical or interventional skills requiring high-grade techniques
Use of cadavers for learning state-of-the-art surgical skills with few chances of OJT, or those difficult to learn through model animals due to their anatomical differences from the human body
4. Research and development of innovative surgical or interventional skills, and medical devices
Use of cadavers for preclinical practice of surgical techniques or developing new surgical devices

These guidelines set out the following four conditions for conducting CST or related activities: (1) the goal is to contribute to the public welfare by improving medical safety through upgrading the surgical skills; (2) it should be implemented as part of medical education or research under the regulations of PECP and the Body Donation Act (BDA); (3) it is essential to have the donors know the details of CST in advance, and IC should be obtained with their ethical, thanatological, and religious beliefs taken into account; and (4) the details of the protocol must be fully scrutinized and approved by the IRB (Table 2).

**Table 2:** Terms and conditions for cadaver use in clinical education and research

1. Any activity that improves and warrants medical safety through clinical education and research, and contributes to social welfare
2. Any activities that are conducted in a framework of medical education and research under the regulations of the PECP and BDA in medical or dental universities
3. Upon the usage of cadavers, the following terms and conditions should be fulfilled, namely: (a) registered donors must have provided a written living will for their body to be used in medical education and research, including education through dissection by anatomical practice by undergraduate students as well as CST by medical doctors or dentists; and (b) if present, the relatives of the deceased person should understand and approve the usage of cadaver for CST
4. The details regarding the conduct of CST must be fully examined and approved by the university’s ethics committee prior to its implementation

## Terms and conditions (Table 2)

We should comply with the following terms and conditions in conducting CST and relative activities:

1. Explicit and reasonable purpose

CST and related activities are aimed at improving medical safety and contributing to social welfare. The details of CST protocol must be carefully reviewed and approved by the IRB (or an equivalent body) in advance, especially its explicit purpose (i.e., clinical education, research, and ethical relevance). Detailed training report, including self-assessment, must be submitted to the IRB after the implementation of the CST. Commercial usage of cadavers or profit-based CST is strongly prohibited, as it violates the philosophy of cadaver donation. All organizers of CST and related activities are required to report the details of the contents, costs, and any conflict of interest (COI) to the JSS CST-PC through the IRB or equivalent body of each institution to guarantee transparency and fairness (Table 3).

**Table 3:** Examples of conflict of interest to be reported

In addition to managing the general conflict of interest (COI) for individual researchers, organizations conducting CSTs and other activities should also report the following to the specialist committee and to the JSS CST PC, to achieve higher levels of transparency in terms of non-profit activity of CST:
1. COI of personnel responsible for conducting and superintending CST
Reporting the COI according to the Japanese Association of Medical Sciences Guidelines for Management of Conflicts of Interest in Medical Research or other guidelines
In case the responsible representative or superintendent is a doctor belonging to a department with an endowed chair or anyone similar, and the COI was raised with the sponsors, this must be explicitly stated
2. COI of institutions (clinical department in universities, academic medical society, research groups or teams, academic seminars, and others) conducting CST and similar activities
In case the participants are charged a fee, the details must be clearly stated
In case donations, sponsorships, or other types of funding have been given by companies, organizations, or individuals, the details must be reported
In case an advertising fee has been received, the details must be reported
In case the medical equipment has been lent by a company, a group, or third-party organization, or if technical support for equipment use, or labor assistance such as installing the equipment has been provided, the details must be reported regardless of charge, the amount paid, and the type of labor provided
3. COI for the research and development activities such as in the industry-academia collaboration
The details of the CST activity in compliance with the guidelines must be reported, along with documentation for the IRB or an equivalent organization

2. Unanimous IC and approval by the registered donor and his/her family

The cadaver donor and his/her family must be informed about the CST, which is different from undergraduate anatomical education. Subsequently, a written IC from the registered donor must be obtained. If the registered donor has family members, their understanding and approval are also mandatory.

3. The department of anatomy fully covers all the administrative routines and handling of cadaver

All the administrative routines from body donation to cadaver management should be centralized and handled by the department of anatomy in the medical or dental school. Having different channels of body donation may bring about misunderstandings and cause trouble with the registered donors and their relatives. Furthermore, participation of any third party must be excluded from the body donation system, which may confront a menace to be misused. This increases the risk of damaging the credit and trust of the body donation system in Japan. Current legal regulations permit us to perform systematic dissection only in specialized dissection rooms in medical or dental schools. Therefore, we strictly follow the regulations to avoid incurring any ethical and moral issues, which lead to losing public trust in the donation system. Cadavers collected by the system or route other than living will is vulnerable to ethical issues. Accordingly, the use of donated cadavers is an essential prerequisite for conducting CST from now on. This guideline does not allow us to use imported cadavers for CST. Furthermore, taking into consideration the ideal environment to uphold the dignity of cadaver, it is hardly possible to carry out CST outside of medical or dental schools or faculties. Therefore, CST must be performed only in such venues.

As a final note, it is important to comply with the guidelines by the JAA so as to avoid delay or interfere with the routine works of the department of anatomy (Comments of the Association of Anatomists on the Guidelines for Human Cadaver Dissection in Clinical Medical Education and Research 2012).

## Points to note

Not only must CST or similar activities be conducted in compliance with the conditions set out above (Table 2), they must also comply with the legal and ethical procedures. The burden of administrative routines on the department of anatomy may be ideally lessened by organizing an expert committee that arranges the consensus and participation of related sections and divisions in the institution.

1. Aim and general conduct
2. The aim of CST and related activities is to contribute to the social welfare by improving medical safety.
3. The details of the CST protocol must be reviewed and checked by the IRB (or equivalent third-party agency) before and after implementation.
4. Participants of CST must honor the wishes of the donor, as well as pay respectful attention in handling the cadaver through deep understanding of the ethical, thanatological, and religious beliefs of Japanese society. At least one representative from among the CST participants is required to attend the memorial service.
5. Participants should take sufficient care to maintain a good relationship between the registered donors or their relatives and the department of anatomy nurtured through a long history of authentic anatomical education.
6. CST must be conducted on a non-profit basis, with clear and transparent accounting. We do not necessarily prohibit charging the participants with a registration fee to join the CST project. Medical devices necessary for conducting CST can be loaned from private companies regardless of charge. Moreover, we can accommodate instruction staff from companies, such as those showing how to use medical equipment safely (Table 3).
7. Administration of donated cadavers and IC
8. The department of anatomy is the sole center for the registration and administration of donor and cadaver, with the record of cadaver stock well-documented.
9. In principle, cadaver donors for CST are requested to have the IC in writing as a living will, with an explicit statement that they wished for their corpse to be used by medical (or dental) doctors for clinical education or research, such as CST.
10. In addition, upon bequeathal of cadaver, the donor’s family should be informed of his/her living will, and their approval should be obtained. This does not apply to those with no family.
11. The whole sequence of administrative procedures such as acceptance, returning and a set of memorial services must be the same as those for the systemic dissection in undergraduate anatomical education.
12. Review of protocol report and disclosure after the conduct of CST
13. Institutions planning to conduct CST or similar activities must have the IRB review the validity and feasibility of the protocol, and give its approval for the implementation.
14. Before the implementation of CST, institutions are required to organize an in-house expert committee, including a member from the department of anatomy, to review the protocols with special references to the aim, methods, number of participants, and the implementation term, before submitting the protocols to the IRB.
15. The protocol for CST or similar activities should indicate the name of the superintending faculty of the department of anatomy and the responsible person from the clinical department.
16. The representative in charge of the implementation of CST should be a professor, an associate professor, or an equivalent medical or dental doctor in the clinical department of the institution concerned. In addition, the representative conducting the training must have an appropriate certification, such as a clinical instructor from the academic society to which he/she belongs.
17. The representative for the implementation of CST and other persons involved in its conduct should report the details of the training (operating costs and COI), whether or not the participants have been charged, and the corresponding fee. If any supply or financial supports are received from third parties such as companies, the details of their involvement (such as the lease of equipment, support for the management costs, sending instructors) must also be reported to the expert committees or equivalent body.
18. Expert committees or equivalent bodies in the institutions should submit a report on the contents of the implementations, including details of costs and the training programs, to the JSS CST-PC in a prescribed format. In case of any surplus or balance carried forward, the reason should be stated clearly.
19. Should the JSS CST-PC have queries on the contents of training, operating costs, or other issues in the report, the Committee may request additional reports from the responsible person in the institution concerned. In addition, if a serious violation of the guidelines is identified, such as conducting training for commercial purposes, the Committee may issue a warning to the parties involved for their non-compliance thereto.
20. The institutions are desirable to disclose the details of CST on their websites.
21. Implementation of CST
22. CST and similar activities should not increase the routine works of the department of anatomy. Those activities should be conducted in specialized facilities in the institutions, such as in anatomy laboratories.
23. The program should devote enough time to expressing respect and acknowledgment to the donor.
24. Cadavers free of chemical fixation require adequate facilities and stringent management, such as frozen storage and infection control, making it mandatory to carry out CST in a suitable institution with good infection management.
25. Liability for accidents during training and how they must be dealt with should be clarified in advance. All participants should acknowledge these before conducting CST.
26. For promoting medical safety on a national scale, the program enables the medical doctors and dentists outside the institutions to take part in the program.
27. Participants’ names and affiliations must be recorded.
